# Inflammatory mechanisms in post-traumatic osteoarthritis: a role for CaMKK2

**DOI:** 10.1097/IN9.0000000000000031

**Published:** 2023-10-16

**Authors:** Keegan C. Riggs, Uma Sankar

**Affiliations:** 1 Department of Anatomy, Cell Biology and Physiology, Indiana University School of Medicine, Indianapolis, IN, USA; 2 Indiana Center for Musculoskeletal Health, Indiana University School of Medicine, Indianapolis, IN, USA

**Keywords:** post-traumatic osteoarthritis, inflammation, chondrocytes, synovium, subchondral bone, Ca^2+^/calmodulin dependent protein kinase kinase 2

## Abstract

Post-traumatic osteoarthritis (PTOA) is a multifactorial disease of the cartilage, synovium, and subchondral bone resulting from direct joint trauma and altered joint mechanics after traumatic injury. There are no current disease-modifying therapies for PTOA, and early surgical interventions focused on stabilizing the joint do not halt disease progression. Chronic pain and functional disability negatively affect the quality of life and take an economic toll on affected patients. While multiple mechanisms are at play in disease progression, joint inflammation is a key contributor. Impact-induced mitochondrial dysfunction and cell death or altered joint mechanics after trauma culminate in inflammatory cytokine release from synoviocytes and chondrocytes, cartilage catabolism, suppression of cartilage anabolism, synovitis, and subchondral bone disease, highlighting the complexity of the disease. Current understanding of the cellular and molecular mechanisms underlying the disease pathology has allowed for the investigation of a variety of therapeutic strategies that target unique apoptotic and/or inflammatory processes in the joint. This review provides a concise overview of the inflammatory and apoptotic mechanisms underlying PTOA pathogenesis and identifies potential therapeutic targets to mitigate disease progression. We highlight Ca^2+^/calmodulin-dependent protein kinase kinase 2 (CaMKK2), a serine/threonine protein kinase that was recently identified to play a role in murine and human osteoarthritis pathogenesis by coordinating chondrocyte inflammatory responses and apoptosis. Given its additional effects in regulating macrophage inflammatory signaling and bone remodeling, CaMKK2 emerges as a promising disease-modifying therapeutic target against PTOA.

## 1. Introduction

Post-traumatic osteoarthritis (PTOA) is a degenerative disease of the joint resulting from trauma. Knee injuries such as anterior cruciate ligament (ACL) tear or meniscal damage are common instigators of PTOA, especially in athletes ^[1,2]^. Impact injuries, another potential cause of PTOA, are commonly seen after trauma sustained from military activities. PTOA pathogenesis involves structural damage from acute injury, pathological mechanical loading, and a residual inflammatory environment in the joint. At the patient level, PTOA causes chronic pain, reduced mobility, and diminished quality of life ^[1–4]^.

Joint trauma results in chondrocyte cell death at the point of injury and fragmentation of the cartilage extracellular matrix (ECM) ^[5]^. Synovial cells respond to this initial damage by producing inflammatory cytokines and reactive oxygen species (ROS) ^[6]^. Inflammatory signaling upregulates matrix-degrading enzymes that further damage the cartilage. Chondrocytes and synovial cells respond to this new damage, propagating a cycle of inflammation and degradation that results in progressive cartilage loss, synovitis, and subchondral bone remodeling ^[7]^.

Articular cartilage is a specialized connective tissue that provides a low-friction surface for the articular joint and facilitates the transfer of mechanical loads ^[8]^. Chondrocytes, the main cell type of cartilage dispersed throughout the ECM, secrete and maintain its components. Cartilage consists mainly of water, type II collagen (COL2), and proteoglycans. It is devoid of nerve innervation, blood vessels, or lymphatics, and has limited intrinsic capacity to repair and regenerate ^[8]^.

The synovium is a specialized connective tissue lining the joint cavity that acts as a seal between the joint fluid and extra-articular connective tissue ^[9]^. Unlike articular cartilage, the synovium has microvascular blood supply, lymphatic vessels, and nerve fibers ^[10]^. The outer layer of synovial connective tissue, rich in type I collagen, is juxtaposed with an infrapatellar fat pad (IFP) such that the synovial membrane and IFP act as an anatomo-functional unit ^[11,12]^. Many studies have highlighted the influence on the function of the synovial membrane elicited by IFP, given the anatomical location of the two structures allowing their close contact. Inflammatory cytokines released into the joint during PTOA stimulate immune cell proliferation and morphological alterations in both tissues ^[12]^.

The inner layer of the synovium is composed of type A and type B synoviocytes lining the joint cavity. Type A synoviocytes are of macrophage lineage, whereas type B synoviocytes are of fibroblast lineage ^[9,10]^. The main function of synovial fibroblasts is to secrete hyaluronic acid and lubricin to maintain the volume and composition of synovial fluid ^[10]^. The synovium in PTOA is characterized by synovial hyperplasia and invasion by leukocytes/lymphocytes from the intravascular compartment ^[9]^. Synovial fibroblasts and resident macrophages are activated to inflammatory states, and circulating monocytes are recruited to the synovium ^[13]^. Synovitis, a hallmark of osteoarthritis (OA), is characterized by synovial fibrosis, macrophage infiltration of the synovium, persistent release of inflammatory cytokines, and angiogenesis ^[9]^. Synovial fibrosis is a nonphysiological wound-healing response characterized by the accumulation of excess fibrous connective tissue in the synovium, contributing to joint pain and stiffness, the main symptoms of OA ^[14]^. Synovial fibrosis is largely found in late-stage osteoarthritis and is potentially induced by pro-inflammatory mechanisms ^[14]^.

Current PTOA treatment focuses on pain management and maintenance of joint function. In the case of mechanical injuries such as ACL rupture, surgical repair provides short-term relief but has not been shown to alter the progression to PTOA ^[15,16]^. There are no current disease-modifying therapies for PTOA. Progression of disease leads to worsening symptoms and increased risk for joint replacement ^[17]^. Recent increased understanding of the signaling pathways involved in PTOA has led to the investigation of new therapeutic targets. In this review, we focus on the inflammatory and apoptotic pathways involved in PTOA and discuss current evidence of Ca^2+^/calmodulin (CaM)-dependent protein kinase kinase 2 (CaMKK2) inhibition as a promising therapeutic strategy.

## 2. PTOA pathogenesis

### 2.1 Pathogenesis—immediate chondrocyte mitochondrial dysfunction and apoptosis following acute trauma

**Figure 1.**
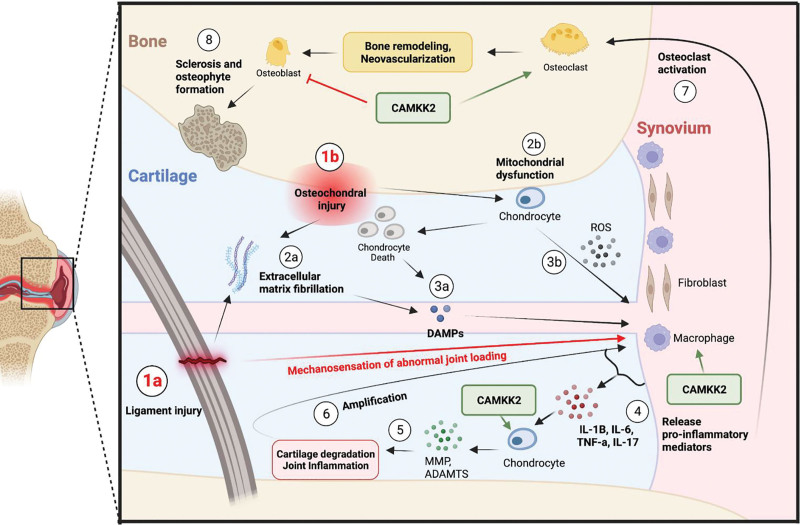
Schematic of PTOA pathophysiology. (**1a**) Ligament injury results in abnormal loading and elevated sheer stress to the joint. Abnormal loading is recognized by synovial macrophages via TRPV-1/4 and Piezo 1/2 mechanoreceptors, thus activating the cells to an inflammatory state. (**1b**) Direct impact causes osteochondral damage with immediate chondrocyte necrosis at high loads. (**2a**) Both impact injury and abnormal loading can cause fragmentation and fibrillation of the cartilage extracellular matrix. (**2b**) Impact injury damages chondrocyte cellular integrity and causes mitochondrial dysregulation. (**3a**) Damage-associated molecular patterns (DAMPs) released from cartilage ECM and from dead chondrocytes are released into the synovium, activating resident macrophages and fibroblasts. (**3b**) Mitochondrial dysregulation causes increased production of reactive oxygen species, which cause chondrocyte apoptosis and activate synovial macrophages. (**4**) Activated synovial cells release inflammatory mediators into the joint space. Key players are IL-1β, IL-6, TNF-α, and IL-17. CAMKK2 is involved in the release of inflammatory cytokines from macrophages. Inflammatory mediators activate more synovial cells to increase inflammatory signaling. These mediators also upregulate the expression of matrix-degrading enzymes in chondrocytes, synovial cells, and bone. CAMKK2 mediates the release of these matrix-degrading enzymes. (**5**) Matrix-degrading enzymes (ie, MMP-3, MMP-13, ADAMTS-4, ADAMTS-5) cause cartilage degradation and joint inflammation. (**6**) Further damage by matrix-degrading enzymes activates even more synovial cells, propagating a cycle of inflammation. (**7**) Pro-inflammatory mediators activate osteoclasts to increase bone resorption and stimulate neovascularization. CAMKK2 stimulates this process. (**8**) Latent TGF-β is released during bone resorption and activates osteoprogenitor cells to increase osteoblasts. Osteoblasts increase bone deposition, resulting in sclerosis and osteophyte formation. CAMKK2 inhibits osteoblast activity. CAMKK2, Ca^2+^/calmodulin-dependent protein kinase kinase 2; ECM, extracellular matrix; IL, interleukin; MMP, markers matrix metalloproteinases; PTOA, post-traumatic osteoarthritis; TNF, tumor necrosis factor alpha; TRPV, transient receptor potential vanilloid-type.

A high-energy impact of the articular surface causes immediate chondrocyte death at the site of the impact and the surrounding cartilage (Figure 1). Early cellular changes occurring in the impacted chondrocytes are characterized by increased mitochondrial electron transport chain (ETC) activity and elevated ROS along with a concomitant decrease in the production of superoxide dismutase in the damaged chondrocytes ^[18–20]^. Free oxygen radicals and metabolites cause cytochrome C to dissociate from the mitochondrial inner membrane, thus activating caspases in the cytosol and initiating apoptosis ^[21]^. Chondroptosis is another type of chondrocyte cell death occurring in vivo, characterized by empty lacunae at the final stage ^[22,23]^. It shares several common features with classical apoptosis such as caspase involvement, cell shrinkage, chromatin condensation, and DNA cleavage, but does not involve phagocytosis ^[22,23]^. Chondrocyte death may also occur via necrosis in areas where immediate trauma causes mitochondrial depolarization beyond a certain threshold ^[19]^. The degree of mitochondrial dysfunction correlates with PTOA severity ^[20]^. Chondrocyte death is positively associated with catabolic markers matrix metalloproteinases (MMP) 1, MMP-3, and MMP-13, and it is negatively associated with levels of COL2 and proteoglycans ^[24]^.

Administration of *N*-acetylcysteine, a free radical scavenger, immediately after chondrocyte impact injury in bovine explants resulted in decreased chondrocyte death and decreased proteoglycan loss days after injury ^[5,25]^. Similar results were observed in bovine explants treated with superoxide dismutase mimetics after impact injury ^[26]^. Immediate treatment with *N*-acetylcysteine or amobarbital, a reversible inhibitor of complex I of the ETC, resulted in decreased chondrocyte apoptosis in a porcine intra-articular fracture model as well as decreased PTOA severity at 6 months after injury ^[5]^. Targeting early mitochondrial dysfunction and ROS formation yielded significant results in these studies, although the time window for efficacy remains unknown.

Nicotinamide adenine dinucleotide phosphate (NADPH) oxidase (Nox) 4 is a ROS-producing protein that is elevated in acute joint trauma. An increase in Nox4 expression was reported in human articular chondrocyte cultures from ACL tear patients and in a murine ACL transection model at 24 and 48 h after injury ^[27]^. Inhibition of Nox4 in these models nullified the excess production of ROS and protected against subchondral bone alterations in vivo. It is unknown if Nox4 inhibition is associated with decreased PTOA development over the long term or if it impacts parameters beyond subchondral bone density.

### 2.2 Pathogenesis—synovial and chondrocyte inflammatory response

Joint injuries and chronic aberrant loading can increase inflammatory signaling in cartilage, synovium, and bone. Inflammatory cytokines, notably interleukin (IL)-6, IL-1β, IL-8, and tumor necrosis factor alpha (TNF-α), are released by chondrocytes and synoviocytes in response to abnormal loading ^[28,29]^. Increase in these cytokines is associated with increased expression of catabolic markers such as MMP-3, MMP-13, a disintegrin and metalloproteinase with thrombospondin motifs (ADAMTS)-4, and ADAMTS-5, which are proteins involved in breaking down collagens and proteoglycans of the ECM. Anabolic markers, such as COL2 and aggrecan (ACAN), decrease correspondingly in the inflammatory phase of PTOA (Figure 1) ^[28,29]^.

Resident macrophages play a key role in the upregulation of MMPs via IL-1β and TNF-α signaling on chondrocytes and macrophages ^[30]^. Multiple mechanisms mediate synovial macrophage activation. This section will discuss toll-like receptor (TLR)-4, nucleotide-binding oligomerization domain, leucine rich repeat and pyrin domain containing (NLRP)3/inflammasome, mechanosensors transient receptor potential vanilloid-type 1/transient receptor potential vanilloid-type 4 (TRPV-1)/TRPV-4, and Piezo ion channels. Synovial fibroblasts also play a role in the release of inflammatory factors, mainly IL-6 ^[31,32]^. Mechanisms in synovial fibroblasts will not be discussed here but are detailed elsewhere ^[31–34]^.

Following trauma or chronic aberrant loading, cartilage fragments are released into the synovium. Damage-associated molecular patterns (DAMPs) from these fragments are sensed by pattern recognition receptors (PRRs) on synovial macrophages ^[28,35]^. For example, the S100A proteins are DAMPs termed “alarmins” released in the joint environment following cartilage damage. In particular, S100A8 and S100A9 are elevated in OA patients and mediate inflammatory changes via TLR4 signaling in macrophages ^[36]^. S100A9-deficient mice were observed to have decreased OA progression ^[37]^. The subsequent release of inflammatory cytokines causes a catabolic shift in chondrocytes and recruits more inflammatory cells to the synovium, propagating the inflammatory response ^[28,35]^. There are multiple other DAMPs and PRRs implicated in OA that have similar PTOA outcomes ^[35]^.

TRPV-1 and TRPV-4 are mechanosensitive cation channels that play a role in the response to articular loading by mediating intracellular calcium transients. In PTOA, these mechanoreceptors are involved in activating macrophages to an inflammatory state ^[38,39]^. Further, TRPV-4 interacts with the NLRP3/inflammasome pathway in macrophages. Accordingly, cartilage-specific knockout of TRPV-4 in mice displays attenuated OA progression ^[39]^. Piezo 1 and Piezo 2 are also mechanosensitive cation channels that transduce excessive mechanical stress responses to articular cartilage chondrocytes and are involved in a Ca^2+^-dependent feed-forward pathogenic mechanism in human OA and PTOA ^[40–43]^. Piezo 1 and Piezo 2 were recently reported to be expressed in the synovial membrane and IFP in human OA, indicating their potential involvement in disease pathogenesis and joint pain ^[44]^.

The inflammasome is a multiprotein complex responsible for upregulating the release of IL-1β, IL-18, TNF-α, and IL-6, and for activating cytosolic caspases, which promote apoptosis. In OA, NLRP3 and NLRP1 are important inflammasome components that trigger this pathological response in macrophages ^[45]^. The inflammasome can be activated in multiple ways, including through PRRs and TRPV-4. Animal models have shown that knockout of NLRP3 does not protect against OA ^[46,47]^. However, increased expression of NLRP3 is associated with OA severity and elevated levels of IL-1β and IL-18 ^[48]^. Therefore, inflammasomes are an attractive therapeutic target against PTOA.

Janus kinase (JAK)/signal transducer and activator of transcription (STAT) is an intracellular signaling pathway downstream of multiple inflammatory cytokines in OA ^[49]^. STAT3 in particular responds to IL-6 and binds the IL-6 promoter in the nucleus, thus creating a positive feedback loop of inflammatory signaling via the IL-6/JAK/STAT3 pathway ^[50]^. In macrophages, the JAK/STAT pathway promotes an inflammatory phenotype. In chondrocytes, the JAK2/STAT3 pathway mediates the effects of IL-6 by upregulating MMP-1, MMP-3, and MMP-13 ^[51]^. Additionally, the JAK2/STAT3 pathway is involved in the reduction of COL2 in chondrocytes ^[52]^. Treatment with a STAT3 inhibitor was shown to decrease the expression of MMP-13 and ADAMTS-7 ^[53]^. JAK inhibitors are food and drug administration (FDA)-approved to treat rheumatoid arthritis ^[54]^, but their efficacy in OA remains unknown. Current investigations focus on unraveling the molecular mechanisms of this pathway and studying relevant therapeutics.

There are multiple anti-inflammatory therapies currently under investigation. Dexamethasone mitigates the response of IL-6 and TNF-α in chondrocytes, thus decreasing the severity of OA ^[55–57]^. Other therapies targeting cytokines include IL-1 receptor antagonist (IL-1Ra), anti-IL-1β, anti-IL-6, and anti-TNF ^[58–63]^. Many of these therapies have shown promising results in animal studies, though only IL-1Ra has been evaluated in clinical trials ^[58]^. A full review of current anti-inflammatory therapeutics approaches for PTOA is discussed elsewhere ^[62]^.

In summary, inflammation in PTOA is characterized by the release of DAMPs from damaged cartilage, activation of resident macrophages to an inflammatory state, recruitment of circulating monocytes to the synovium, production of inflammatory cytokines by these activated cells, and upregulation of matrix-degrading enzymes in chondrocytes (Figure 1). In humans, there is a persistent inflammatory response in the joint regardless of restored joint biomechanics ^[15,16,64,65]^. Therefore, targeting inflammation is a promising strategy for treating PTOA.

### 2.3 Pathogenesis—subchondral bone remodeling

Although OA is classically a disease of the cartilage, the subchondral bone is also affected by sclerosis and osteophyte formation. Subchondral bone alteration directly affects stiffness and strain in the diseased joint, but it also has indirect effects on the cartilage through crosstalk.

Microfractures in the subchondral bone cause uncoupling of bone remodeling. An initial osteoclast-dominant phase decreases bone thickness and increases porosity. These changes trigger the release of latent TGF-β, which activates osteoprogenitor proliferation and stimulates neovascularization. A resulting increase in osteoblast activity promotes sclerosis and osteophyte formation ^[66–68]^. Elevated TGF-β in OA models is associated with increased subchondral bone disease, and inhibition of TGF-β decreases disease ^[69,70]^. These effects are local at the subchondral bone. Systemically, TGF-β acts as an anti-inflammatory molecule and protects against OA ^[71]^. Due to these reasons, TGF-β is a less promising therapeutic target in OA. Subchondral bone pathology is also associated with early mitochondrial dysfunction, with IL-1β playing a role in the process. Intra-articular injection of IL-1Ra and Nox4 after intra-articular fracture in animal models decreased the severity of subchondral bone sclerosis and osteophyte formation ^[27,59]^.

Prostaglandin E2 (PGE2), a product of the cyclo-oxygenase 2 (*COX-2*) gene, is secreted by osteoblasts, chondrocytes, and macrophages in an inflammatory state. PGE2 is elevated in the subchondral bone in OA, leading to subchondral bone alteration and pain hypersensitization via E prostanoid 4 (EP4) receptors ^[72,73]^. Inhibition or deletion of EP4 attenuates subchondral bone sclerosis in mouse models. This is relevant to current clinical therapy since COX-2 inhibitors, such as nonsteroidal anti-inflammatory drugs are routinely used to alleviate pain. The expression of inflammatory mediators by chondrocytes is thought to be influenced in part by osteoblasts. The subchondral bone in animal models of PTOA was found to have increased levels of IL-6, nitric oxide (NO), and MMPs ^[74]^. Subchondral bone osteoblasts increase the expression of matrix-degrading proteins via extracellular signal-regulated kinase (ERK)1/2 and phosphoinositide 3-kinase/a serine/threonine kinase (PI3K/AKT) signaling pathways ^[75–78]^.

Subchondral bone alterations and osteophyte formation still occur in patients despite restoration of joint mechanics ^[64,79]^. Therapeutically targeting osteoblasts and osteoclasts could thus be beneficial to halting PTOA. More research is needed to fully understand the crosstalk between bone and cartilage in the progression of the disease.

## 3. CaMKK2 as a therapeutic target against OA

As previously discussed, inflammatory cytokines, such as IL-1β and TNF-α, are elevated following aberrant mechanical stress in the joint ^[28,29]^. In chondrocytes, these cytokines result in the phosphorylation of phospholipase C gamma, which stimulates Ca^2+^ release from the endoplasmic reticulum via interaction with tubulin ^[80]^. Ca^2+^ functions as a second messenger regulating a variety of intracellular processes. While transient increases in intracellular Ca^2+^ are vital to cell function, its sustained elevation following its release from dysfunctional mitochondria is associated with chondrocyte apoptosis, cartilage catabolism, and decreased cartilage anabolism ^[81]^.

Transient increases in intracellular Ca^2+^ are immediately sensed by calmodulin (CaM), and the Ca^2+^/CaM complex binds to and activates a plethora of downstream proteins including members of the Ca^2+^/CaM-dependent protein kinase (CaMK) signaling cascade. The binding of Ca^2+^/CaM allows for the activation of the upstream serine/threonine protein kinases CaMKK1 and CaMKK2 through autophosphorylation, which then phosphorylates the downstream kinases CaMKI and CaMKIV ^[82]^. CaMKK2 uniquely regulates cellular responses to metabolic stress via the phosphorylation and activation of adenosine mono phosphate-dependent protein kinase, through a Ca^2+^/CaM-dependent mechanism ^[82]^. The absence of CaMKK2 in macrophages protects against inflammation by mitigating their response to TLR4 stimulation ^[83]^. Additionally, absence of CaMKK2 in mice caused elevated bone mass due to decreased osteoclast and increased osteoblast activity ^[84]^. With influence on both inflammation and bone remodeling, CaMKK2 emerged as a promising target against PTOA.

CaMKK2 levels and activity were elevated in the cartilage of mice that underwent destabilization of the medial meniscus (DMM) surgery to induce PTOA, and in primary murine articular chondrocytes treated with IL-1β ^[85]^. Genetic deletion or pharmacological inhibition of CaMKK2 conferred protection against PTOA in vivo. IL-1β-induced upregulation of IL-6 and PGE2/COX-2 as well as MMP-13 and ADAMTS-5, were attenuated in CaMKK2-deficient chondrocytes in vitro and following DMM surgery, indicating that CaMKK2 acts downstream of IL-1β in PTOA. In addition, CaMKK2 deletion counteracted the anti-anabolic effects of IL-1β with observed preservation of COL2 and ACAN levels compared with those in wild-type mice that underwent DMM surgery. CaMKK2-deficient chondrocytes treated with IL-1β had lower phosphorylated STAT3 levels compared with wild-type ^[85]^. Since STAT3 is known to upregulate MMP-13 and ADAMTS-5, these studies indicate that CaMKK2 signaling mediates inflammation in chondrocytes via the IL-1β/IL-6/STAT3/MMP-13 pathway ^[86]^.

Synovitis is also influenced by CaMKK2 as its deletion or inhibition in mice leads to diminished synovial inflammation and macrophage infiltration after DMM surgery ^[85]^. These results are consistent with the known role of CaMKK2 in macrophage inflammatory response ^[83]^. Subchondral bone sclerosis was also absent in DMM mice after CaMKK2 deletion or inhibition, indicating protection against PTOA.

A recent study highlighted the potential role of CaMKK2 in human primary OA ^[87]^. Similar to observations from the mouse PTOA model, CaMKK2 mRNA and protein were elevated in human OA cartilage compared with paired intact cartilage from the same femoral head collected from total hip arthroplasty surgical discards. Increased CaMKK2 was associated with increased expression of MMP-13 and decreased expression of anabolic markers COL2 and ACAN in the diseased cartilage ^[87]^. Overexpression of intact CaMKK2 but not a functionally defective mutant in human chondrocytes elevated the levels of MMP-13, pSTAT3, and the pro-apoptotic marker BCL2 associated X (BAX), indicating that CaMKK2 enzyme activity is required for its role in coordinating inflammatory signaling in chondrocytes. Further, pharmacological inhibition of CaMKK2 activity suppressed chondrocyte death in OA cartilage explants ^[87]^. Findings from this study indicate that CAMKK2 regulates catabolic and apoptotic responses in human primary chondrocytes, potentially through STAT3/MMP-13 and BAX-mediated mechanisms, further bolstering the status of CaMKK2 as a promising therapeutic target in the treatment of established primary OA, which currently has no clinical disease-modifying treatments.

## 4. Conclusion and perspectives

PTOA is a degenerative disease of the joint resulting from acute trauma or adverse loading following ligamentous injury. The disease pathogenesis is multifactorial, including cartilage degradation, synovial inflammation, and subchondral bone remodeling. Current treatment options focus on surgical restoration of joint mechanics and management of pain. Though these therapies provide short-term relief, they are not disease-modifying. In a clinical setting, early OA tends to be asymptomatic, and many patients do not seek treatment until they have developed advanced disease. For this reason, ideal therapeutic interventions would target both early and late mechanisms in disease pathogenesis.

Pathogenesis differs in PTOA resulting from acute injury to the chondral surface and chronic adverse loading after ligamentous injuries. Acute mechanical damage to the joint is mediated in part by mitochondrial dysregulation, production of ROS, and chondrocyte death at the site of the injury. This damage also triggers inflammation at the synovium and chondrocytes and later leads to subchondral bone remodeling. Chronic aberrant loading causes upregulation of inflammatory cytokines and catabolic markers leading to global joint alteration. In addition, there is crosstalk between the cartilage, synovium, and subchondral bone that further exacerbates inflammation and cartilage catabolism. Many therapeutics targeting mitochondrial dysfunction, inflammatory signaling, and catabolic responses are currently under investigation. One such target is CaMKK2 as its inhibition modulates cell death, inflammation, and catabolism in animal and human OA. It has protective effects on the cartilage, synovium, and subchondral bone, though further investigation is needed to understand the exact mechanisms by which CaMKK2 affects these tissues. Current human studies have been performed on samples with existing osteoarthritis, so the effect of CaMKK2 inhibition on OA pathogenesis in humans is yet to be observed. Figure 1 summarizes the key events leading to PTOA and the potential role of CaMKK2 in modulating this disease.

Given that different mechanisms are at play in early versus late PTOA, future research comparing immediate and delayed treatment windows is needed. This is clinically relevant as patients present at different stages of disease progression. Future studies comparing routes of administration and frequency of dosing are also needed to test the efficacy of the treatment and its feasibility in a clinical setting.

## Author contributions

K.C.R.: Design, investigation, writing-original draft preparation, revising and editing. U.S.: Conceptualization, writing, reviewing, editing, supervision, funding acquisition, and project administration. K.C.R. and U.S. have read and approved the article.

## Conflicts of interest

The authors declare they have no conflict of interest.

## Funding

This study was supported by National Institutes of Health grant 5R01AR076477—CaMKK2 Signaling in Osteoarthritis and an Investigator-Initiated Research Award W81XWH-20-1-0304 from the US ARMY MEDICAL RESEARCH ACQUISITION ACTIVITY (to Dr. Sankar). K.C.R. was supported by a Comprehensive Musculoskeletal T32 Training Program from the NIH (AR065971). The study sponsors were not involved in any aspects of this study including study design, collection, analysis, and interpretation of data; or in the writing of the article, and in the decision to submit the article for publication. The content is solely the responsibility of the authors and does not necessarily represent the official views of the National Institutes of Health.

## Acknowledgments

The authors thank the funding sources (NIH R01-AR076477, DoD-IIRA W81XWH-20-1-0304 and NIH Training grant AR065971) for supporting this work.
